# Influenza-induced microRNA-155 expression is altered in extracellular vesicles derived from the COPD epithelium

**DOI:** 10.3389/fcimb.2025.1560700

**Published:** 2025-05-27

**Authors:** Laura V. Reid, C. Mirella Spalluto, Tom M. A. Wilkinson, Karl J. Staples

**Affiliations:** ^1^ Clinical & Experimental Sciences, University of Southampton Faculty of Medicine, Southampton, United Kingdom; ^2^ National Institute for Health Research (NIHR) Southampton Biomedical Research Centre, University Hospitals Southampton NHS Foundation Trust, Southampton, United Kingdom

**Keywords:** COPD, influenza, extracellular vesicle, microRNA, epithelium

## Abstract

**Background:**

Influenza virus particularly affects those with chronic lung conditions such as Chronic Obstructive Pulmonary Disease (COPD). Airway epithelial cells are the first line of defense and primary target of influenza infection and release extracellular vesicles (EVs). EVs can transfer of biological molecules such as microRNAs (miRNAs) that can modulate the immune response to viruses through control of the innate and adaptive immune systems. The aim of this work was to profile the EV miRNAs released from bronchial epithelial cells in response to influenza infection and discover if EV miRNA expression was altered in COPD.

**Methods:**

Influenza infection of air-liquid interface (ALI) differentiated BCi-NS1.1 epithelial cells were characterized by analyzing the expression of antiviral genes, cell barrier permeability and cell death. EVs were isolated by filtration and size exclusion chromatography from the apical surface wash of ALI cultured bronchial epithelial cells. The EV miRNA cargo was sequenced and reads mapped to miRBase. The BCi sequencing results were further investigated by RT-qPCR and by using healthy and COPD primary epithelial cells.

**Results:**

Infection of ALI cultured BCi cells with IAV at 3.6 x 10^6^ IU/ml for 24 h led to significant upregulation of anti-viral genes without high levels of cell death. EV release from ALI-cultured BCi cells was confirmed using electron microscopy and detection of known tetraspanin EV markers using western blot and the ExoView R100 platform. Differential expression analyses identified 5 miRNA that had a fold change of >0.6: miR-155-5p, miR-122-5p, miR-378a-3p, miR-7-5p and miR-146a-5p (FDR<0.05). Differences between EV, non-EV and cellular levels of these miRNA were detected. Primary epithelial cell release of EV and their miRNA cargo was similar to that observed for BCi. Intriguingly, miR-155 expression was decreased in EVs derived from COPD patients compared to EVs from control samples.

**Conclusion:**

Epithelial EV miRNA release may be a key mechanism in modulating the response to IAV in the lungs. Furthermore, changes in EV miRNA expression may play a dysfunctional role in influenza-induced exacerbations of COPD. However, further work to fully characterize the function of EV miRNA in response to IAV in both health and COPD is required.

## Introduction

Chronic obstructive pulmonary disease (COPD) causes significant morbidity and mortality worldwide (Adeloye et al., 2015). It is a major social and economic burden with patients typically having impaired quality of life that deteriorates considerably with exacerbation frequency (Zhou et al., 2015). COPD exacerbations are associated with increased airway inflammation usually triggered by bacterial and viral infections (Kwak et al., 2016). Recognition of the importance of viral infection in COPD exacerbations has increased over recent years due to the development of technology for the detection of respiratory viruses (Yin et al., 2018). Exacerbations triggered by viral infections are usually associated with hypersusceptibility of greater airway inflammatory response, more severe symptoms, and delayed recovery compared to those without viral infections (Tu et al., 2021).

Influenza is one of the most common viruses associated with COPD exacerbations (Mohan et al., 2010). Recently influenza has been reported to significantly increase the risk of ischemic stroke, pneumonia, respiratory failure, as well as COPD acute exacerbations among COPD patients (Liao et al., 2022). Although the host response is essential for viral clearance, in some cases, influenza A virus (IAV) induces excessive inflammation that can be detrimental to the host via the amplification of tissue damage and increased susceptibility to fatal secondary infection (Tavares et al., 2017). This inflammatory response has been widely studied in terms of cytokines, chemokines and other soluble mediators. However, in recent decades, research into the role of extracellular vesicles (EVs) in modulating disease pathology has increased dramatically.

EVs are highly heterogeneous lipid bilayer particles produced by most cell types. Based on the mechanism of biogenesis, EVs can be categorized into three distinct subgroups; exosomes, microvesicles and apoptotic bodies (Théry et al., 2018). Exosomes are the smallest subclass of EVs with a diameter ranging from 30 nm to 100 nm (Johnstone et al., 1987). They are formed as intraluminal vesicles by inward budding of endosomal membrane and are then released into the extracellular space (Kowal et al., 2014). Several molecules are involved in the biogenesis of intraluminal vesicles, such as the Endosomal Sorting Complex Required for Transport machinery, lipids (such as ceramide) and the tetraspanins (such as CD9, CD63, CD81) (Kowal et al., 2014).

Originally considered to be cell debris, EVs have since been shown to transfer lipids, proteins and nucleic acids as a form of intercellular communication (Simons and Raposo, 2009; Raposo and Stoorvogel, 2013). EV cargo varies depending on the cellular origin or pathophysiological conditions. Comprehensive ‘omics’ approaches including proteomic (mass spectrometry (MS)) and transcriptomic (RNA sequencing) analysis have successfully enabled profiling of EV cargo. The role of EVs has been widely implicated in many chronic lung diseases (Trappe et al., 2021). This study focuses on the role of microRNA (miRNA) as EV cargo.

There are a growing number of studies that report isolation and characterization of lung-derived EVs from bronchoalveolar lavage fluid (BALF) (Burke et al., 2022). Airway epithelial cells and alveolar macrophages are thought to be the major producers of EVs in the lung (Kulshreshtha et al., 2013; Soni et al., 2016). Using cell type-specific membrane tagging and single vesicle flow, Pua et al. reported that 80% of murine lung-derived EVs were of epithelial origin (Pua et al., 2019). Given their ability to transfer cargo it is possible airway epithelial EVs may be an important mechanism in modulating the anti-viral immune response (Hiemstra et al., 2015). However, it is extremely difficult to ascertain the impact of infection in samples from the human lung, such as bronchoalveolar lavage (BAL) fluid or sputum and so a relevant *in vitro* model is needed.

This study investigates the microRNA cargo of extracellular vesicles released from bronchial epithelial cells following infection with IAV. To do this we have established, for the first time, an air-liquid interface (ALI) bronchial epithelial cell model for IAV infection and isolation of EVs using a relevant cell line and primary bronchial epithelial cells (PBECs). We have compared the EV miRNA released from IAV infected bronchial epithelial cells to non-infected control using small RNA Sequencing and identified changes in specific EV miRNAs released from infected cells. Finally, we have compared the EV miRNA released from healthy and COPD primary epithelial cells in response to influenza A virus.

## Methods

### Subjects

We recruited ex-smoking subjects with mild to moderate COPD (as per GOLD) (n=3) and healthy ex-smoking volunteers (HV-ES) (n=3), all with ≥10-pack year history and had stopped smoking ≥6 months prior to enrolment, as part of the MICAII study (**Table 1**). Patients with a history of asthma or atopy were excluded. All subjects gave written informed consent, and the study was approved by National Research Ethics Service South Central ethical standards – Hampshire A and Oxford C Committees (LREC no: 15/SC/0528). Sampling was undertaken using fiberoptic bronchoscopy and epithelial brushings were recovered and processed as previously described (Watson et al., 2020).

**Table 1:** Volunteer demographics.

Subject/sample characteristics	COPD (n=3)	Healthy ex-smoker (n=3)	P value
Age, median (IQR)	66 (52-69)	53 (45-72)	0.500
Male, (%)	100	100	–
Smoking pack years, median (IQR)	39 (35-52)	26 (22-30)	0.050
BMI, median (IQR)	28.9 (27.4-32.4)	26.3 (24.8-34.3)	0.350
Lung Physiology			
FEV1 (% predicted), median (IQR)	67 (66-88)	97 (78-104)	0.100
FVC (% predicted), median (IQR)	98 (85-104)	97 (80-106)	0.500
FEV1/FVC%, median (IQR)	59 (50-69)	77 (74-79)	0.050

BMI, body mass index; FEV1, forced expiratory volume in 1 sec, FVC, forced vital capacity; IQR, interquartile range. Mann-Whitney test.

### Bronchial epithelial cell culture

Immortalized human bronchial epithelial cells (BCi-NS1.1), a kind gift of Prof Ron Crystal (Cornell, New York (Walters et al., 2013)) were defrosted after storage at -80°C and maintained in PneumaCult™-Ex Plus Medium (Stemcell Technologies, Cambridge, UK – details of all media used are in **Table 2**). BCi cells were cultured in a T75 flask coated with 1:10 dilution of PureCol^®^ Type I Bovine Collagen (Advanced BioMatrix, California, USA) at 37°C and 5% CO2. Cells were expanded until 70% confluence and passaged with trypsin-EDTA solution 1X (Sigma-Aldrich, Poole, UK). The cells were pelleted by centrifugation at 400 g for 5 minutes and seeded in a collagen coated T75 flask. Cells were used between passages 20-25.

**Table 2:** PneumaCult media final compositions.

Reagent	Final Composition
Pneumacult-Ex Complete Medium	*PneumaCult-Ex Basal Medium (Stemcell Technologies)* *Pneumacult-Ex Supplement 1X (Stemcell Technologies)* *Hydrocortisone 1X (Stemcell Technologies)* *0.125 μg/ml amphotericin B (Sigma-Aldrich)* *25 μg/ml gentamicin (Gibco, Paisley, UK)*
Pneumacult-Ex Plus Complete Medium	*PneumaCult-Ex Basal Medium (Stemcell Technologies)* *Pneumacult-Ex Plus Supplement 1X (Stemcell Technologies)* *Hydrocortisone 1X (Stemcell Technologies)* *0.125 μg/ml amphotericin B (Sigma-Aldrich)* *25 μg/ml gentamicin (Gibco)*
Pneumacult-ALI Complete Medium	*PneumaCult-ALI Basal Medium (Stemcell Technologies)* *Pneumacult-ALI Supplement 1X (Stemcell Technologies)* *Pneumacult-ALI Maintenance Supplement 1X (Stemcell Technologies)* *Hydrocortisone 1X (Stemcell Technologies)* *Heparin Solution (Stemcell Technologies)* *100 U/ml penicillin (Sigma-Aldrich)* *100 μg/ml streptomycin (Sigma-Aldrich)*
Pneumacult-ALI Infection Medium	*45 ml PneumaCult-ALI basal media* *5 ml PneumaCult-ALI supplement* *100 U/ml penicillin (Sigma-Aldrich)* *100 μg/ml streptomycin (Sigma-Aldrich)* *0.02% Bovine Serum Albumin (BSA) (Sigma-Aldrich)*
Pneumacult-Ex Infection Medium	*45 ml PneumaCult-Ex Basal Medium* *100 U/ml penicillin (Sigma-Aldrich)* *0.02% Bovine Serum Albumin (BSA) (Sigma-Aldrich)*

PBECs were isolated from bronchial brushes and expanded using PneumaCult™-Ex Plus Medium, (Stemcell) in T75 flasks coated with collagen (PureCol). When cells were confluent, they were trypsinized and transferred to ALI culture as below.

Air-liquid interface (ALI) cultures were generated by seeding submerged cultured cells at 100,000 cells/insert for 24 well plates on PureCol^®^ Type I Bovine Collagen-coated Transwell permeable polyester membrane inserts (Corning Costar, High Wycombe, UK) with a 0.4 μm pore size. Pneumacult™-Ex Plus Complete Medium was added to both the apical and basal compartments. After 5 days, the media was replaced in apical and basal compartments with Pneumacult™-ALI Medium (Stemcell) prepared according to the manufacturer’s instructions. After a further 2 days the media was removed from the apical chamber. The Pneumacult™-ALI Medium was replaced in the basal compartment every 2–3 days and mucus was washed off the cells every week during the differentiation period. ALI cultures were maintained for around 28 days until a pseudostratified epithelium was observed. Differentiation was confirmed by the observation of a homogeneous layer of cells and presence of cilia using a Leica DM-IL microscope (Leica, Milton Keynes, UK) with phase-contrast and a transepithelial electrical resistance (TEER) greater than 500 Ω cm2 measured using a Millicell ERS-2 Voltohmmeter (Merck Millipore, Watford, UK).

### Extracellular vesicle isolation

EVs were recovered from ALI apical secretions by two sequential washes as follows. HBSS (Sigma-Aldrich) was added to the apical compartment, incubated for 30 min at 37°C and 5% CO_2_ and then collected. Samples were then centrifuged at 300 g for 10 min. The supernatant was collected and centrifuged at 1200 g for 20 min and then filtered through a 0.22 µm PES sterilized filter (Merck Millipore), to remove any larger particles. The sample was then loaded onto an equilibrated Amicon^®^ Ultra-15 (10,000 MWCO) spin filter (Merck Millipore) and centrifuged at 3200 g, 4°C for 15 min to further purify and concentrate the sample. The filter device was washed with DPBS (Sigma-Aldrich) and centrifuged at 3200 g, 4°C for at least a further 15 min or until the sample was concentrated.

Separation of EVs based on size was completed using PURE-EVs™ size exclusion columns (HansaBioMed Life Sciences^®^, Tallinn, Estonia). Prior to use, columns were washed with 30 ml of DPBS to eliminate any preservative buffer residues. Up to 2 ml of the concentrated sample was then added to the column. In total, 24 fractions each of 500 µL in volume were collected. The initial fractions contain the void volume in which buffer within the column prior to sample application is eluted. EVs are the first particles to be eluted at around fractions 6–11 as has previously been confirmed by the manufacturer. Based on the manufacturer’s instructions and previous work completed by Burke et al, EVs have been demonstrated to be eluted in fractions 6-11 (elution volume 2.5-5.5 ml) (Burke et al., 2022). Fractions were then combined to form 4 groups as follows; fractions 1–5 termed SEC#1, fractions 6–11 termed SEC#2, fractions 12–17 termed SEC#3 and fractions 18–24 termed SEC#4 (Burke et al., 2022). These grouped fractions were concentrated using an equilibrated Amicon^®^ Ultra-4 (10,000 MWCO) spin filter, centrifuged at 3200 g, 4°C for at least 30 min. The sample was then recovered from the filter device and stored at -80°C for analyses.

### Influenza infection

Differentiated ALI culture cells were washed with Hanks’ Balanced Salt solution (HBSS) to remove any apical mucus (Sigma-Aldrich) and fresh PneumaCult-ALI Basal Medium containing 1 x PneumaCult-ALI supplement (Stemcell) plus 100 U/ml penicillin, 100 μg/ml streptomycin and 0.02% Bovine Serum Albumin (BSA) (all Sigma-Aldrich) was added to basolateral side. Influenza A/Wisconsin/67/2005 (H3N2, Virapur, San Diego, CA) was supplied at a TCID50 (Tissue Culture Infectious Dose required to kill 50% of cells) of 3.6 x 10^8^ IU/ml (infectious units/ml) and diluted in HBSS and 50 µl of diluted virus was applied apically at a TCID50 of 3.6 x 10^6^ IU/ml (multiplicity of infection (MOI) 0.3). An uninfected control with HBSS added apically was also completed. Cells were then incubated at 37°C and 5% CO_2_ for 2 h. After this time the apical side was washed twice with HBSS to remove any excess virus or mucus. The cells were then incubated at 37°C and 5% CO_2_ for a further 22 h. Infected cells were collected at 24 h for qPCR analyses in 1 ml of QIAzol (Qiagen, Manchester, United Kingdom) and stored at -80°C. Apical washes including any mucus produced were collected at 24 h for analysis.

### Measurement of protein concentration

Protein concentration was determined by the Pierce BCA Protein Assay kit (Thermo Fisher Scientific^®^, Basingstoke, UK) according to the manufacturer’s instructions. The absorbance was measured at 550 nm using a ThermoMax Microplate Reader (Molecular Devices, Berkshire, UK). The average 550 nm absorbance measurement of the Blank standard replicates was subtracted from the 550 nm measurement of all the other standards and unknown sample replicates. A standard curve was plotted using the Blank-corrected 550 nm measurement for each BSA standard versus the concentration (µg/ml). This was then used to determine the concentration of each unknown sample.

### LDH assay

Cell death was analyzed by the release of Lactate Dehydrogenase (LDH) into supernatants using the CytoTox 96^®^ Non-Radioactive Cytotoxicity Assay (Promega, Southampton, UK) following the manufacturer’s instructions. Briefly, 50 μl of the CytoTox 96^®^ Reagent was added to 50 μl of cell supernatant or cell lysate then incubated in the dark for 30 min at room temperature. Following this incubation, 50 μl of Stop Solution was added to each well and the absorbance was measured at 490 nm using a ThermoMax Microplate Reader (Molecular Devices).

### RNA isolation

RNA was isolated from cells and SEC elution samples using miRNeasy Micro kit (Qiagen^®^) according to the manufacturer’s instructions. Briefly, 1 ml of QIAzol Lysis Reagent (Qiagen^®^) was added directly to cells or concentrated 200 μl SEC elution sample. The samples were then stored at -80°C. Once accumulated the samples were then defrosted on ice and 200 μl of chloroform (Sigma) was added. The samples were shaken vigorously for 15 seconds and incubated at room temperature for 5 min. Samples were then centrifuged for 15 min at 12,000 g at 4°C. The upper aqueous phase was combined with 1.5 volumes of 100% ethanol then added in an RNeasy MinElute spin column in a 2 ml collection tube. The samples were then centrifuged in RNeasy MinElute spin columns at 8000 g for 15 seconds at room temperature. For SEC elution samples DNase digestion was completed according to Appendix B of the miRNeasy Micro kit protocol for samples containing <1µg total RNA. The sample was then rinsed with 700 μl of Buffer RWT then 500 μl Buffer RPE by centrifugation at 8000 g for 15 seconds. Then 500 μl of 80% ethanol was added to the RNeasy MinElute spin column and centrifuged for 2 min at 8000 g. The spin column was then centrifuged at 12,000 g for 5 min to dry the membrane and 14 μl of RNase-free water was added and centrifuged at 12,000 g to elute RNA. Concentrations of RNA were determined by NanoDrop 1000 spectrophotometer (Thermo Fisher Scientific^®^).

### RT-PCR

Synthesis of cDNA was carried out using Taqman Advanced miRNA cDNA synthesis kit (Applied Biosystems) according to the manufacturer’s instructions. The cDNA was then diluted 1:10 with RNase free water (Sigma-Aldrich). The real-time quantitative polymerase chain reaction (RT-qPCR) was performed using a PCR reaction mix containing 2.5 µl TaqMan Universal Master Mix II no UNG (Applied Biosystems), 1.25 µl RNase-free water and 0.25 µl TaqMan Advanced miRNA Assays (Applied Biosystems) (**Table 3**). 1 μl of diluted cDNA was combined with 4 μl of a PCR reaction mix and the RT-qPCR was performed using a 7900HT Fast Real-Time PCR System (Thermofisher). The reaction mix was incubated at 95°C for 10 min to activate enzyme and then completed 40 cycles of denaturing at 95°C for 15 seconds and annealing/extension at 60°C for 1 min. MicroRNA miR-26b-5p was identified as a suitable endogenous control as it was both the highest and most stably expressed miRNA across both IAV and uninfected BCi EVs in our small RNASeq analysis.

**Table 3:** TaqMan Assay IDs (Applied Biosystems).

Assay Name	Assay ID
hsa-miR-16-5p	477860_mir
hsa-miR-24-3p	477992_mir
hsa-miR-138-5p	477905_mir
hsa-miR-182-5p	477935_mir
hsa-miR-26b-5p	478418_mir
hsa-miR-155-5p	483064_mir
hsa-miR-122-5p	477855_mir
hsa-miR-146a-5p	478399_mir
hsa-miR-378a-3p	478349_mir
hsa-miR-7-5p	483061_mir
B2M	Hs00984230_m1
ACTB	Hs99999903_m1
FLUWISCONSIN15	AP47VVA
CXCL10	Hs00171042_m1
SOCS1	Hs00705164_s1
IFNB	Hs01077958_s1
ISG15	Hs01921425_s1

The FLUWISCONSIN15 primer was a custom designed primer that was previously used in reference 20.

### Transmission electron microscopy

Transmission electron microscopy (TEM) was completed as previously reported (187). Briefly, 5 μL of EVs in 1X PBS was layered onto individual formvar-carbon coated 200 mesh copper grids (Agar Scientific Ltd, Stansted, UK). After 1 min, the grid was blotted to remove excess liquid. The samples were then contrasted in a solution of 5% ammonium molybdate (w/v) plus 1% trehalos. TEM micrographs were obtained with the FEI Tecnai T12 instrument at Biomedical Imaging Unit, University Hospital Southampton with an 11-megapixel side mounted camera (Morada^®^ G2, EMSIS Ltd, Muenster, Germany).

### Western blotting

Particles from SEC eluted samples were lysed by adding 10X RIPA (abcam) to a final concentration of 1X. Samples were vortexed and incubated on ice for 30 min. Cells were lysed following removal of the culture media and a wash with DPBS. Lysis was completed by adding 1 ml of 1X RIPA (ThermoFisher Scientific) combined with Halt Protease inhibitor (ThermoFisher Scientific) to the apical face. Cells were then incubated on ice for 15 min before collection into a 1.5 ml microcentrifuge tube. Samples were then stored at -80°C. Reduced sodium dodecyl sulphate-polyacrylamide gel electrophoresis (SDS-PAGE) was conducted using NuPAGE^®^ 12% Bis-Tris Protein Gels (Invitrogen, Paisley, UK) according to the manufacturer’s instructions. Analyses of CD9, STCH and calnexin was completed under reducing conditions with NuPAGE^®^ LDS Sample Buffer (Invitrogen) and NuPAGE^®^ Reducing Agent (Invitrogen) added to samples. Analyses of CD63 was completed under non-reducing conditions with NuPAGE^®^ LDS Sample Buffer added to the samples. The samples were incubated at 70°C for 10 min. The sample was then centrifuged for 10 min at 12,000 g and loaded onto the protein gel alongside 5 µl of PageRuler Prestained Protein Ladder (ThermoFisher Scientific). Electrophoresis was completed at 200 V (110mA) for 50 min. Proteins were transferred to a Polyvinylidene difluoride (PVDF) membrane using an iBlot Dry Blotting System with an iBlot Transfer Stack (PVDF) (Invitrogen), according to the manufacturer’s instructions. Membranes were blocked in Tris-buffered saline (TBS) with 0.1% Tween-20 (wash buffer) containing 5% (w/v) skimmed milk for 1 h rocking at room temperature. Primary antibodies CD9 (ab92726, abcam), CD63 (ab59479, abcam), STCH (ab127750, abcam) and calnexin (C5C9, cell signaling) diluted 1:1000 were applied separately to the membrane and incubated overnight. Membranes were then washed three times with wash buffer before being incubated with relevant secondary antibody either horse radish peroxidase-conjugated goat α-mouse IgG antibody (ab205719) or horse radish peroxidase-conjugated goat α-rabbit IgG antibody (ab205718) diluted 1:2000. The membranes were then washed three times with wash buffer. To visualize the proteins, SuperSignal™ West Pico PLUS Chemiluminescent Substrate (Thermo Scientific) was used with reagents mixed in a 1:1 ratio and then applied to the surface of the membrane. A ChemiDoc MP Imaging System (BioRad, Hertfordshire, UK) was then used to detect the luminescent signal visualized using ImageLab software.

### Apolipoprotein E ELISA

The presence of contaminating lipoproteins was determined using a Human Apolipoprotein E (ApoE) Enzyme-Linked Immunosorbent Assay (ELISA) (ab108813) (abcam, Cambridge, UK) as per the manufacturer’s instructions. Briefly, standards or test samples were added to the wells of an anti-ApoE antibody 96-well plate. Subsequently, an ApoE specific biotinylated detection antibody was added and washed with the provided wash buffer. Streptavidin-Peroxidase Complex was then added and 3,3′,5,5′-Tetramethylbenzidine (TMB) was used to generate a reaction with the colorimetric signal quantified by ThermoMax Microplate Reader.

### ExoView analysis

Single particle interferometric imaging measurement was performed by NanoView Biosciences using the ExoView R100 platform. CD9, CD63 and CD81-positive EVs were immunocaptured on a tetraspanin microarray chip and imaged as single particles. Particle size was analyzed using single particle interferometric reflectance imaging sensing (SP-IRIS) using the ExoView Human Tetraspanin Kit (NanoView Biosciences, Malvern, UK). Co-expression of tetraspanin proteins were then assessed by labelling the captured EVs with a cocktail of fluorescence antibodies conjugated to CD81-Alexa555, CD63-Alexa647 and CD9-Alexa488. The chips were then imaged with the ExoView R100 reader with sizing thresholds set to 50-200nm diameter. A 150-μm-diameter area of each capture spot was selected for analysis using an automated circle finding algorithm. The particles within this area were counted, producing a particle value that represents normalization of particle count to spot area. Each chip has the antibody capture spots in triplicate.

### RNA sequencing

RNA isolated for miRNA sequencing was completed by Qiagen using the miRNeasy Mini Kit (Qiagen) according to manufacturer’s instructions. Briefly, EVs (suspended in 150 μl of 1X DPBS) were lysed using QIAzol Lysis Reagent (Qiagen). To assess the quality of RNA isolation across samples, QIAseq miRNA Library Quality control (QC) Spike-Ins (Qiagen) were added to each of the lysed EV samples. RNA was extracted, using phenol/chloroform-based phase separation and silica membrane–based purification, with an elution volume of 14 μl. The library preparation was performed by Qiagen using the QIAseq miRNA Library Kit (Qiagen) as per the manufacturer’s instructions. A total of 5 μl total RNA was converted into miRNA Next Generation Sequencing (NGS) libraries. Briefly, library preparation included adapter ligation, reverse transcription with the introduction of Unique Molecular Index’s (UMIs), library amplification using PCR (22 cycles), addition of sample indices and sample purification. Library preparation was quality controlled using capillary electrophoresis (Agilent DNA 1000 Chip). The libraries were then pooled in equimolar ratios, based on quality and concentration measurements, and then single end reads of 75 nucleaotides in length were sequenced (average 20 million reads/sample) by Qiagen on a NextSeq (Illumina Inc.) sequencing instrument according to the manufacturer instructions.

The FASTQ files generated were analyzed by Qiagen using FastQC, a quality control tool for high throughput sequencing data. Read processing was carried out by Qiagen using CLC Genomics Server 21.0.4. In summary, the reads were processed by trimming the common sequence, UMI and adapters. In addition, reads with length < 15 nt or > 55 nt were filtered out and deduplicated using the UMI. Reads were grouped into UMI groups when they start at the same position based on the end of the read to which the UMI is ligated, are from the same strand or have identical UMIs. Groups that contain only one read (singletons) are merged into non-singleton groups if the singleton’s UMI can be converted to a UMI of a non-singleton group by introducing a single nucleotide polymorphism (SNP). Trimmed read length distributions demonstrated that all samples have a peak at the expected miRNA read length of around 21 nucleotides. Qiagen mapped the reads to human miRBase version 22 using the workflow “QIAseq miRNA Quantification” of CLC Genomics Server with standard parameters. Reads were also mapped by Qiagen to the human piRNA database hsa.v1.7.6. All reads that did not map to miRBase or piRNA database were mapped by Qiagen to the human genome GRCh38 with ENSEMBL GRCh38 version 97 annotation. This was carried out using the “RNA-Seq Analysis” workflow of CLC Genomics Server with standard parameters.

Unsupervised filtering, data analysis and differential expression analysis was performed in Southampton using RStudio^®^, using R (v 4.1.1). The methods were adapted from the Bioconductor package, “Empirical analysis of digital gene expression in R” (edgeR). Lowly-expressed miRNA were filtered out given that they are likely to below minimal level to have biological effect or may interfere with downstream analysis. A conservative value of 10 counts in a minimum of 5 samples was chosen as a cut-off margin for filtering to ensure maximum differential expressed miRNA were captured. The filtered data was normalized using “Trimmed Mean of M-values” normalization (TMM) implemented in the calcNormFactors function in the edgeR package (Andersen et al., 2004; Robinson et al., 2010). Differential expression analysis was completed using edgeR and EV miRNAs that were most stably expressed across all samples from the miRNA sequencing results were also investigated using NormFinder software (Andersen et al., 2004).

### Bioinformatic analysis of miRNA function

*In silico* miRNA target network analyses was conducted using miRNet 2.0 (www.mirnet.ca) and miRNA gene target database miRTarBase (https://bio.tools/mirtarbase). MiRNet was used to visualize the complex underlying networks between the miRNA and their target genes. The functional roles of the miRNA target gene network were also investigated in miRNet using Kyoto Encyclopedia of Genes and Genomes (KEGG) pathway analysis, where p-values <0.05 were regarded as statistically significant for the functional analysis. Functional analyses of miRNA target genes identified by miRTarBase was also completed using ToppFun function of ToppGene an online biological information database that performs KEGG pathway analysis (https://toppgene.cchmc.org/enrichment.jsp).

### Statistical analysis

Analysis of two groups was performed using a Mann-Whitney U test if unpaired or Wilcoxon’s signed rank test if paired. For more than two groups, Kruskal-Wallis (unpaired) or Friedman (paired) ANOVA with a Dunn’s multiple comparisons test were used (GraphPad Prism v10, GraphPad Software Inc., San Diego, USA). Results were considered significant if p<0.05.

## Results

### Characterization of EVs from influenza-infected BCi cells

**Figure 1:**
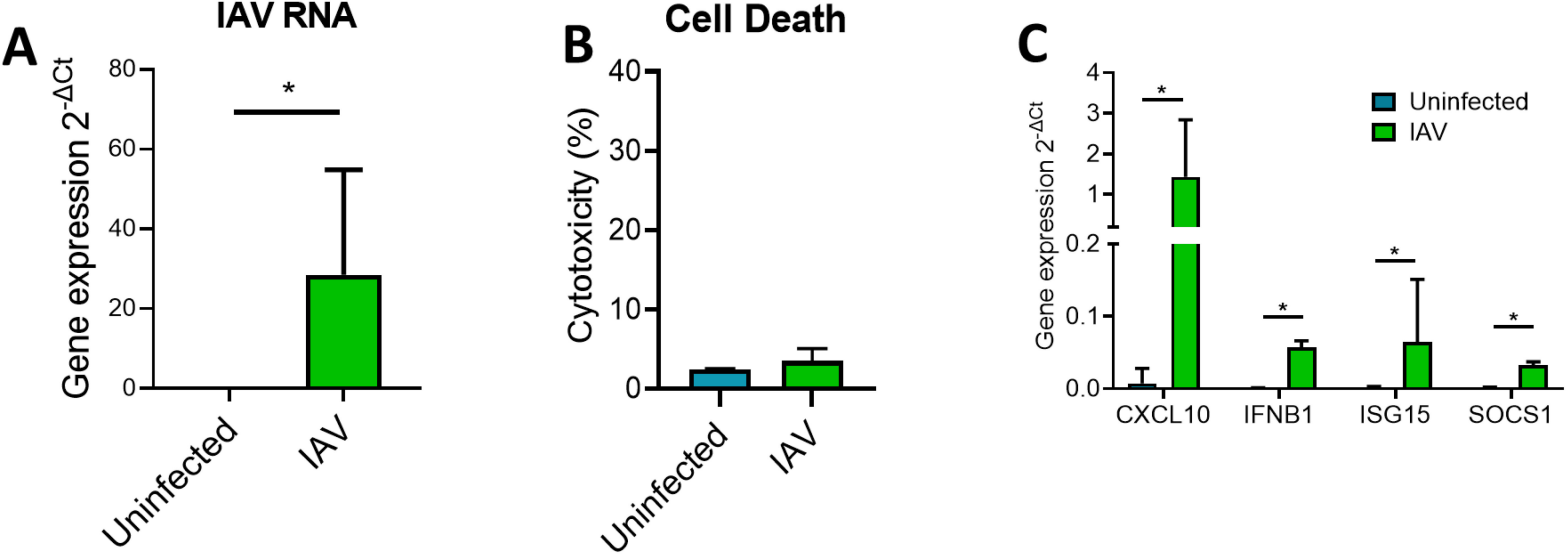
ALI BCi infection with IAV is associated with increased inflammatory gene expression. Analysis was completed for IAV infected BCi (3.6 x 10^5^ IU/ml) and uninfected BCi 24h. **(A)** The intracellular level of viral RNA encoding IAV HA was analyzed via qPCR. ΔCT values of viral RNA was calculated as Ct values of IAV HA minus Ct value of housekeeping gene (*ACTB*). **(B)** Cytotoxicity was calculated as LDH release as a percentage of the total LDH measured following cell lysis (n=6). **(C)** The expression of *CXCL10*, *IFNB1*, *ISG15* and *SOCS1* was analyzed via qPCR. ΔCT values were calculated from the Ct values of gene of interest minus Ct value of housekeeping gene (*ACTB*). Fold change was calculated for IAV infected sample compared to uninfected sample. Normality determined by Shapiro-Wilk test. Data displayed (n=6) with median (IQR) and analyzed using Wilcoxon test. *P<0.05.

We first used the immortalized BCi-NS1.1 cell line to establish a robust methodology to isolate EVs from ALI cultures following influenza infection. ALI-cultured BCi infected with IAV at 3.6 x 10^6^ IU/ml for 24 h demonstrated a significant increase in the detection of HA mRNA (**Figure 1A**). This increase in viral detection was associated with no significant increases in LDH release indicating minimal cell death at this time point (**Figure 1B**). Moreover, this increase in virus was accompanied by significant increases in the expression of innate immune response genes (**Figure 1C**).

**Figure 2:**
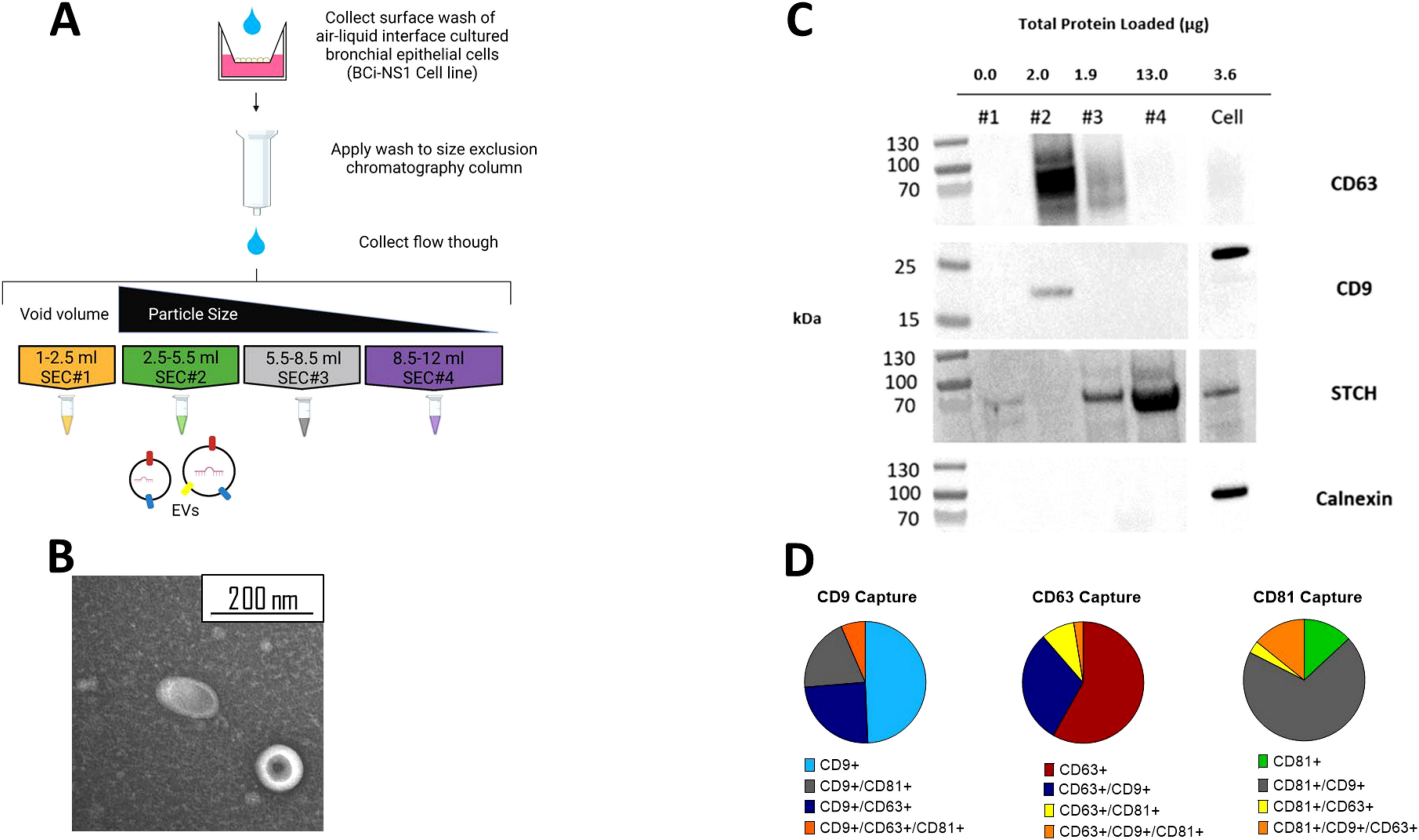
EVs separated from other soluble protein using SEC. **(A)** Schematic of SEC EV isolation protocol from ALI BCi. **(B)** TEM image of SEC#2 isolated samples from apical wash of ALI BCi demonstrating cup-shaped morphology. **(C)** Presence of EV proteins (CD63 and CD9) and absence of non-EV proteins (STCH and Calnexin) detected in SEC#2 sample. Left lane contains prestained protein ladder with protein size indicated in kDa. **(D)** ExoViewR100 demonstrates proportion of particles captured by either CD9, CD63, CD81 antibodies. Average based on three technical replicates.

Size-exclusion chromatography (SEC) was then used to isolate EVs from the apical wash of the ALI cultures (**Figure 2A**). One of the main limitations of SEC is that EVs have been shown to be co-isolated with lipoprotein particles due to a similarity in size (Brennan et al., 2020). To confirm the absence of lipoprotein released from ALI BCi an ApoE ELISA was performed. ApoE was not detected in the SEC filtrate nor in the concentrated apical wash prior to isolation (data not shown). A signal was detected from the positive control, suggesting that this result was due to either no or extremely low concentrations of ApoE being released by BCi cells and not an error in the experimental technique. To provide further confirmation that EVs were present in the isolated sample, fractions were visualized using TEM. Visualization of grouped SEC fraction SEC#2 revealed particles with the expected size (<200 nm) and “cup shaped” morphology previously observed for EVs under TEM (**Figure 2B**).

Western blot analyses was used to further demonstrate the presence of EVs and lack of contaminating particles and other molecules in SEC#2 isolated samples from ALI BCi cultures (**Figure 2C**). SEC fraction and cell lysates were loaded onto the same gel. The same volume of each of the different SEC fractions was loaded. The tetraspanin, CD63, commonly used as an EV marker was predominantly detected in SEC#2 and to a lesser extent in SEC#3. Tetraspanin CD9, also commonly used as an EV marker, was only detected in SEC#2. In addition, the molecular weight of CD9 was lower for SEC#2 compared to the cell lysate. There was no evidence of the endoplasmic reticulum protein calnexin in any of the SEC fractions.

To further characterize the EV population, 200 µl of concentrated SEC#2 sample was sent for analyses by NanoView Biosciences using the ExoView R100. A 200 µl non-isolated sample concentrated from 10 ml of apical wash prior to filtration or SEC was also analyzed. This technology allows quantification of all tetraspanin positive particles using fluorescent antibodies against the tetraspanins. This analyses showed highest concentration of particles in both the isolated and non-isolated samples to be CD9 positive followed by CD63 and CD81 (**Figure 2D**). Epithelial cells released a heterogenous population of EVs with around half of CD9+ particles only containing CD9+, with the remainder containing two tetraspanins and only a small percentage containing all three. CD63+ particles again predominantly only contained one tetraspanin however CD63+ is more commonly found co-isolated with CD9 compared to CD81. Lastly, CD81+ particles predominantly also contained CD9. A similar amount of particles contained only CD81 or all three tetraspanins with little co-localization observed with just CD63.

### Analysis of microRNA content of EVs released from influenza-infected BCi cells

**Figure 3:**
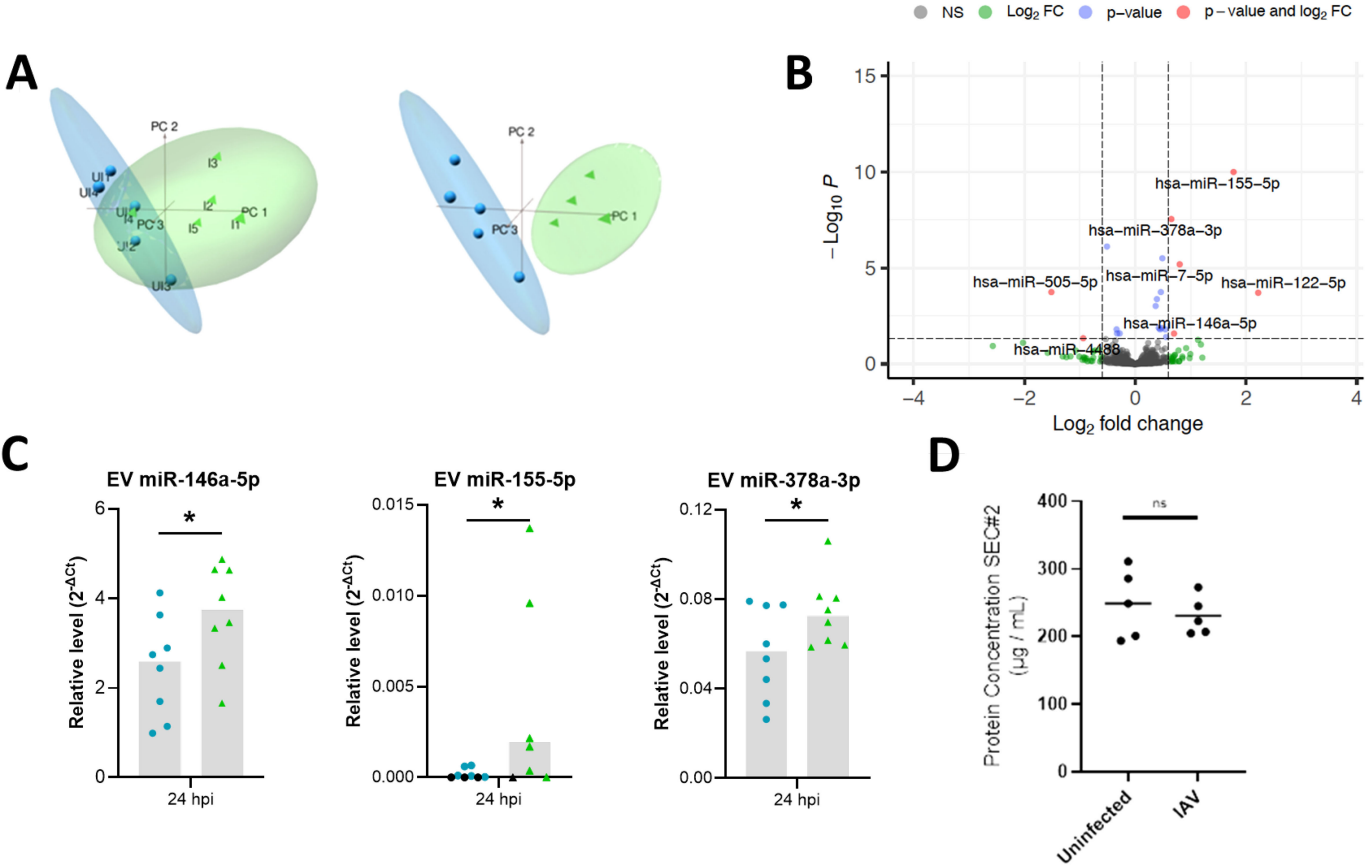
Differential expression analyses reveals miRNA altered in IAV-infected BCi EVs. **(A)** Principal component analysis (PCA) plots of filtered and normalized for miRbase using TMM normalization prior to (left) and following (right) removal of infected EV outlier. Samples are EVs isolated from uninfected ALI BCi (Blue) and EVs isolated from IAV infected ALI BCi (green). **(B)** Volcano plot displaying log_2_FC and log_10_ FDR of differentially expressed miRNA with logFC > 0.6 or logFC< -0.6. **(C)** Relative level of miR-146a-5p, miR-155-5p and miR-378a-3p measured using qPCR compared to miR-26b-5p for EV isolated from uninfected or IAV infected BCi at 24 h post infection (n=8). Bar graph shows median. Black data points=Not detected. **(D)** Protein concentration of SEC#2 fraction for uninfected and IAV infected ALI. Bar indicates median (n=5). Wilcoxon’s test. *=P<0.05.

Reads obtained from miRNA sequencing of uninfected (n=5) and IAV infected (n=5) BCi EVs were mapped to the mature miRNA database (miRBase). The average proportion of reads mapped to miRBase was 31.8% for EVs released from uninfected BCi and 34.8% for EVs released from IAV-infected BCi. To visualize the sources of variation in the data principal component analysis (PCA) was used. PCA of TMM normalized miRBase mapped data reveals separate clustering across PC1 of uninfected and infected BCi EVs based on miRNA except for one influenza data point (I4) that appears to cluster with the uninfected data. The influenza datapoint (I4) was therefore determined to be an outlier and removed from the dataset for differential expression analysis (**Figure 3A**).

Differential expression analysis of uninfected and IAV-infected BCi EVs was then performed and the results summarized in a volcano plot (**Figure 3B**). 6 miRNA met the log_2_FC 0.6 cut-off to identify miRNA altered above the minimal level of change of miRNA previously reported to have a significant impact on the biology of the cell (Kok et al., 2018). Of these, 5 miRNA were identified to be upregulated for IAV-infected BCi EVs with logFC > 0.6 (miR-122-5p, miR-155-5p, miR-146a-5p, miR-7-5p, miR-378a-3p) and 1 downregulated with logFC < -0.6 (miR-505-5p).

The expression of these EV miRNA was then validated using RT-PCR. Of the 6 miRNA identified as being differentially-expressed after 24 h influenza infection by RNA Sequencing, only miR-146, miR-155 and miR-378 were significantly increased by infection when assessed by RT-PCR (**Figure 3C**). Importantly, infection with influenza did not appear to affect the yield of EVs as there was no significant difference in the protein concentrations between the SEC#2 fraction derived from infected or uninfected cultures (**Figure 3D**).

### Characterization of EVs from influenza-infected PBECs

**Figure 4:**
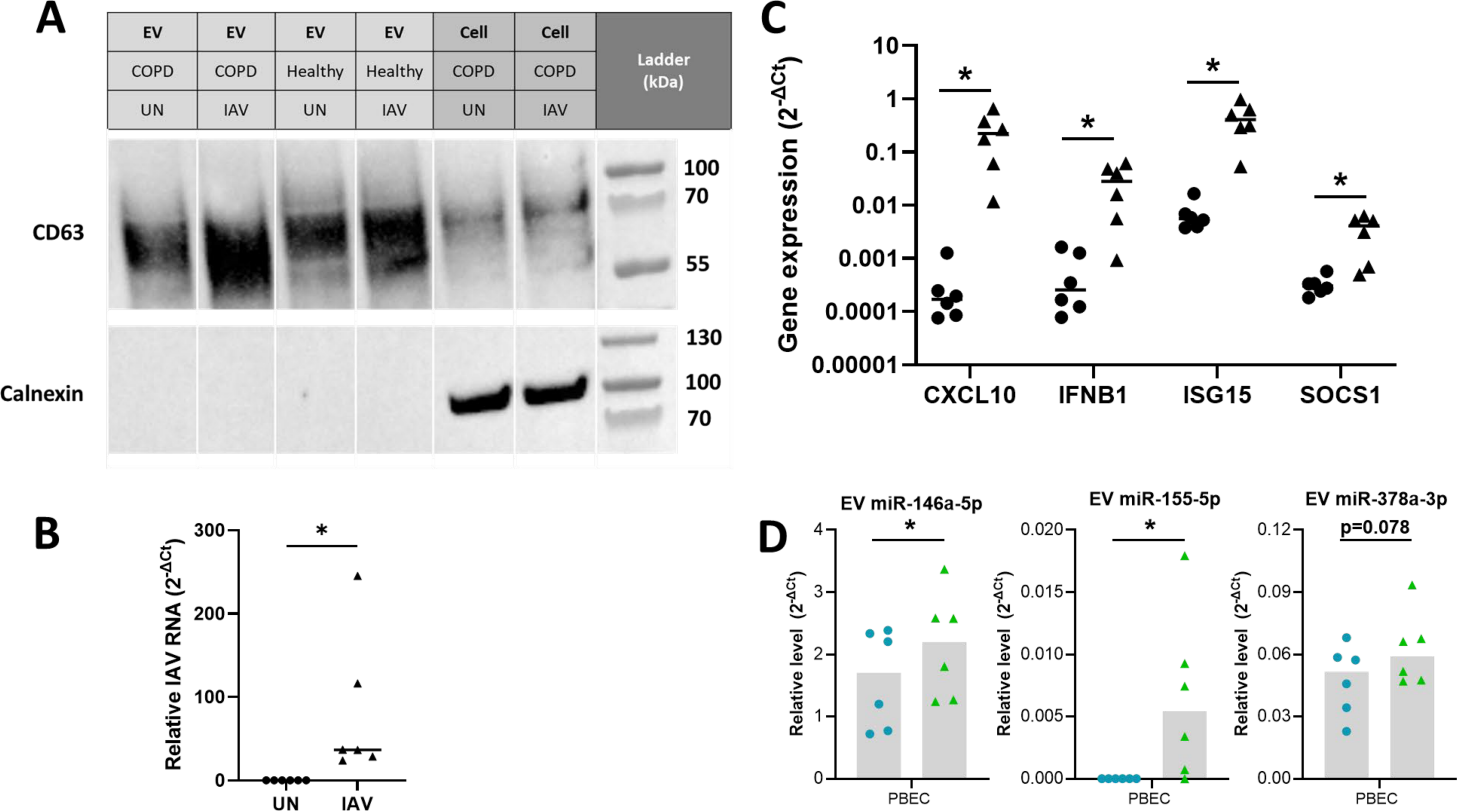
Differential expression analyses reveals miRNA altered in IAV-infected PBEC EVs. **(A)** Presence of EV proteins (CD63 and CD9) and absence of non-EV proteins (STCH and Calnexin) detected in SEC#2 samples derived from cultured PBEC from both control and COPD volunteers. Left lane contains prestained protein ladder with protein size indicated in kDa. **(B)** The intracellular level of viral RNA encoding IAV HA was analyzed via qPCR. ΔCT values of viral RNA was calculated as Ct values of IAV HA minus Ct value of housekeeping gene (ACTB). **(C)** The expression of *CXCL10*, *IFNB1*, *ISG15* and *SOCS1* was analyzed via qPCR. ΔCT values were calculated from the Ct values of gene of interest minus Ct value of housekeeping gene (ACTB). Fold change was calculated for IAV infected sample compared to uninfected sample. **(D)** Relative level of miR-146a-5p, miR-155-5p and miR-378a-3p measured using qPCR compared to miR-26b-5p for EV isolated from uninfected or IAV infected BCi at 24 h post infection. Normality determined by Shapiro-Wilk test. Data displayed (n=6) with median and analyzed using Wilcoxon test. *P<0.05.

To validate that these influenza-induced changes were not limited to the BCi cell line, influenza-infection of PBEC cultured at ALI was then investigated in PBEC from healthy (n=3) and COPD donors (n=3). Uninfected and IAV-infected EV samples from healthy and COPD PBEC were confirmed to be enriched in CD63 EV marker while non-EV protein calnexin was shown to be absent (**Figure 4A**). PBEC were able to be infected with IAV (**Figure 4B**) and infection caused an increase in expression of immune genes including *CXCL10*, *IFNB1*, *ISG15* and *SOCS1* (**Figure 4C**). The expression of miRNAs in EVs from the PBECs was then investigated using RT-PCR (**Figure 4D**). Similarly to the BCi cells, influenza infection caused significant upregulation of EV miR-146 and miR-155 expression, whilst there was only a trend towards an increase in miR-378 expression.

### miR155 expression is decreased in PBEC EVs derived from COPD patients

**Figure 5:**
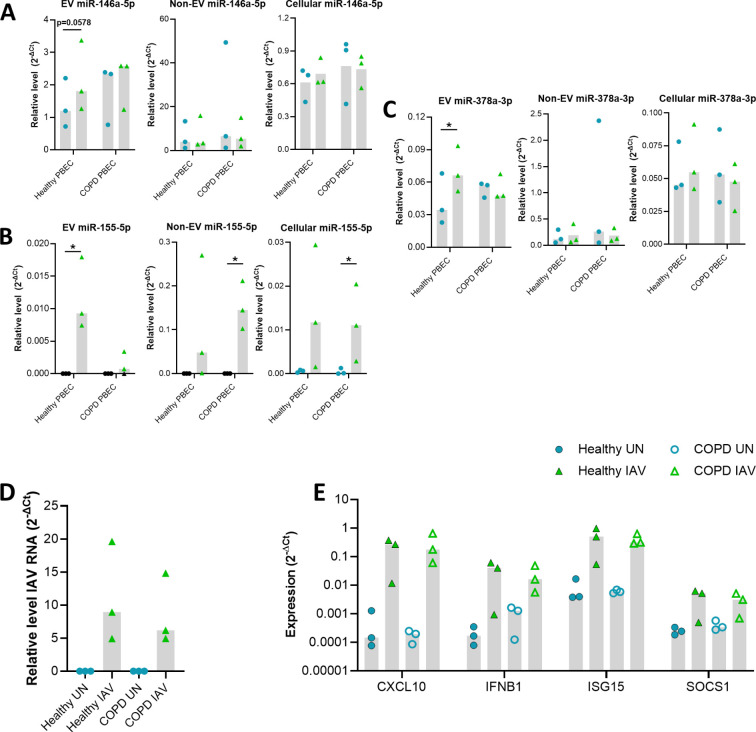
Validation of miRNA altered in IAV infected control and COPD PBECs via qPCR. Relative level of **(A)** miR-146a-5p, **(B)** miR-155-5p and, **(C)** miR-378a-3p compared to miR-26b-5p for EV, Non-EV and cellular samples from COPD (n=3) and control (n=3) PBECs uninfected or IAV infected at TCID50 of 3.6 x 10^6^ for 24 hours. **(D)** The intracellular level of viral RNA encoding IAV HA was analyzed via qPCR. ΔCT values of viral RNA was calculated as Ct values of IAV HA minus Ct value of housekeeping gene (*ACTB*). **(E)** The expression of *CXCL10*, *IFNB1*, *ISG15* and *SOCS1* was analyzed via qPCR. ΔCT values were calculated from the Ct values of gene of interest minus Ct value of housekeeping gene (ACTB). Fold change was calculated for IAV infected sample compared to uninfected sample. Blue circle= Uninfected, Green triangle=infected. Black data points=Not detected. Normality determined by Shapiro-Wilk test. Bar graph displays median and analyzed using a Friedman ANOVA and Dunn’s multiple comparison test. *=P<0.05.

These influenza-induced changes were then investigated to ascertain if there were different responses in epithelial cells derived from COPD patients compared to healthy controls (**Figures 5**). An increase in the relative levels of miR-146a-5p, miR-155-5p and miR-378a-3p was detected for healthy EVs but not COPD EVs in response to IAV (**Figure 5A-C**), although there was only a significant difference in the relative levels of EV miR-155-5p expressed between healthy and COPD samples (Mann-Whitney p=0.05). In contrast, increased relative levels of miR-155 were detected for non-EV (SEC#3/4) and cell samples (**Figure 5B**). On the other hand, no significant difference in the relative levels of miR-146a-5p or miR-378a-3p were detected for both healthy and COPD non-EV and cell samples in response to IAV.

Despite this difference in EV miR-155-5p expression between health and COPD, there was no significant difference in the levels of IAV RNA detected between healthy and COPD PBECs (**Figure 5D**). Furthermore, there did not seem to be any impact of this change in EV miR-155-5p expression on the expression of the immune genes we measured (**Figure 5E**). The expression of *CXCL10*, *IFNB1*, *ISG15* and *SOCS1* was similar between healthy and COPD PBECs.

### Bioinformatic analyses of function of target genes of EV miRNA altered in response to IAV

**Figure 6:**
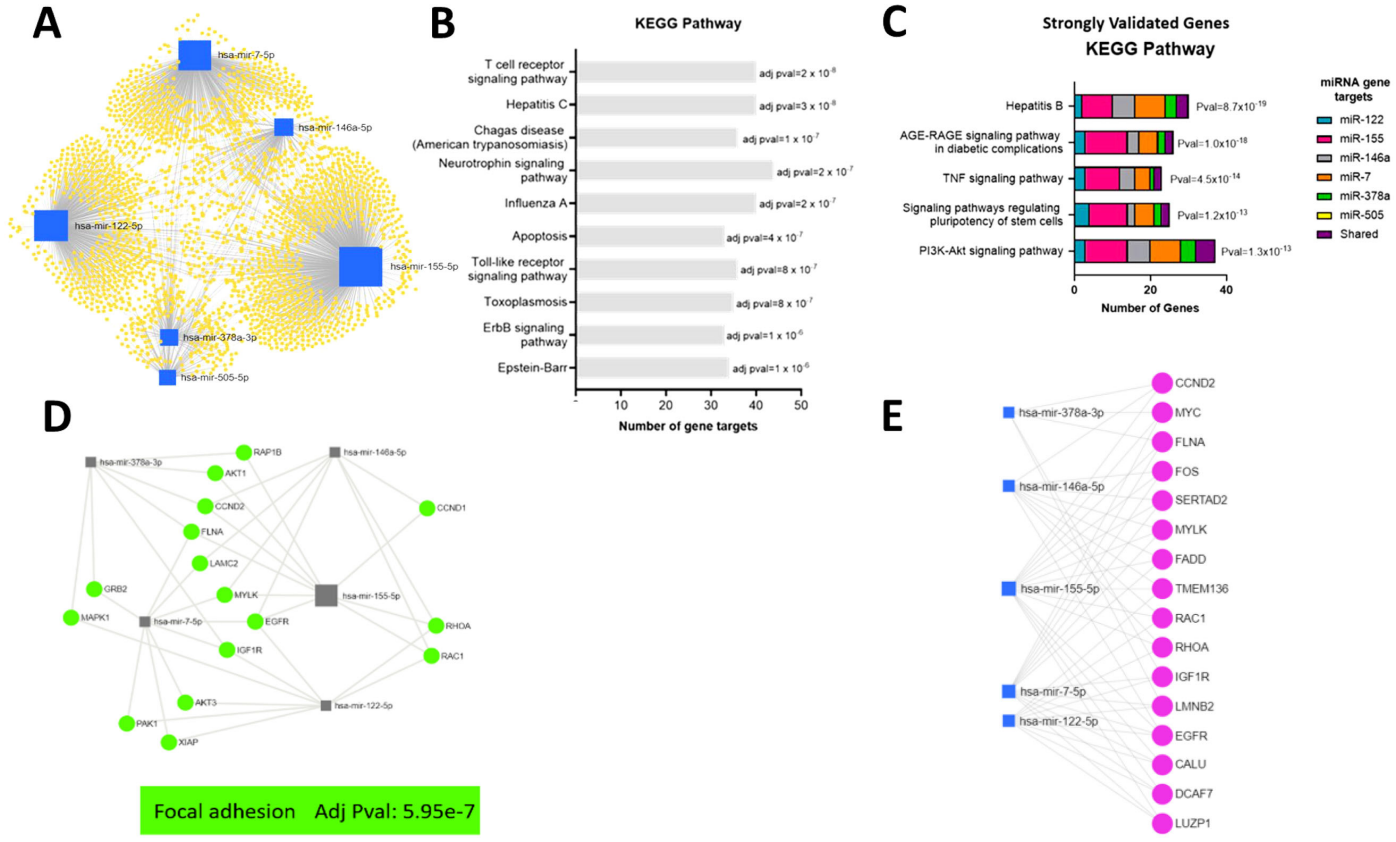
Bioinformatic analyses of function of target genes of EV miRNA altered in response to IAV. **(A)** A network of all potential target genes of the significantly different miRNA (miR-505-5p, miR-378a-3p, miR-7-5p, miR-146a-5p, miR-155-5p and miR-122-5p) were identified using miRTarBase v8.0 and visualized and functionally analyzed in miRNet. **(B)** Top ten KEGG pathways associated with target genes based on miRNet analyses without cancer terms. **(C)** Top KEGG pathways associated with reporter assay verified target genes identified using miRTarBase v8.0. **(D)** Top Focal Adhesion KEGG pathway genes associated with verified target genes based on miRNet analyses of genes targeted by at least two miRNA. **(E)** Top verified genes targeted by at least three miRNA.

To further understand the functional relationship between the EV miRNA and response to IAV, correlation of expression of viral RNA or anti-viral immune genes and miRNA was investigated (**Figure 6A**). Given that the purpose of this research was to investigate the viral response and that cancer terms are over-represented in curated bioinformatic datasets, cancer terms were removed from the KEGG analyses (**Figure 6B**). Reassuringly, many of the top KEGG pathways identified in this analysis were viral pathways including Hepatitis C, Influenza A and Epstein-Barr. In addition, there were several KEGG pathways related to immune response including T cell receptor signaling and toll-like receptor signaling. Furthermore, apoptosis also appears in the top KEGG pathways. Genes enriched in the top KEGG pathways were predominantly targeted by miR-155 and miR-7. This finding can at least partially be attributed to the fact these miRNAs are amongst the most widely studied and therefore have more identified gene targets.

To increase the confidence of the *in silico* functional analyses of miRNA target genes, the list of target genes for EV miRNA altered in response to IAV (miR-122-5p, miR-155-5p, miR-146a-5p, miR-7-5p, miR-378a-3p and miR-505-5p) was reduced to those validated by reporter assays. The biological function of the gene targets validated by reporter assays were then investigated using the KEGG pathway analysis function of the ToppFun platform (**Figure 6C**). Interestingly, as observed when investigating all the target genes, the hepatitis viral pathway appears as one of the top KEGG pathways (excluding cancer terms). Furthermore, genes in this pathway remain predominantly targets of miR-155 and miR-7. Other top KEGG pathways were AGE-RAGE signaling pathway, TNF signaling pathway, signaling pathway regulating pluripotency of stem cells and PI3K-Akt signaling pathway. Some of these previously validated target genes were shared targets for more than one of the miRNAs altered in EVs in response to IAV. The shared gene targets of the miRNA upregulated in EVs in response to IAV were therefore investigated. This was completed in miRNet using all the target genes identified by miRTarBase. Filters were then applied to select target genes associated with at least two miRNA or at least three miRNAs. The biological function of the gene targets was then investigated using the KEGG pathway analysis function.

The top KEGG pathway for genes targeted by at least two miRNA was focal adhesion (**Figure 6D**). Focal adhesions are specialized structures in which many of the biological responses to external forces are originated. These large multiprotein complexes mechanically link the extracellular matrix to the cytoskeleton via integrin membrane receptors (Wolfenson et al., 2013). Furthermore, miRNet identified 16 genes to be targeted by three or more of the upregulated miRNA (**Figure 6E**). Only one gene, *EGFR*, was targeted by 4 miRNA and no genes were targeted by greater than 4 miRNA. *EGFR* regulates focal adhesion and cellular responses to the environment (Rao et al., 2020). Genes targeted by at least 3 miRNA were also identified to be involved in focal adhesion (**Figure 6E**).

## Discussion

The presence of RNAs, including miRNAs, within EVs was identified almost 2 decades ago (Valadi et al., 2007). Since then studies have demonstrated EV miRNA, released from cells *in vitro* or obtained from biofluids, are altered in response to a range of stimuli including viral infection (Chahar et al., 2018; Othumpangat et al., 2021). Furthermore it is now widely accepted that miRNA transferred by EVs can induce functional effects in a recipient cell (Chen et al., 2017). This work has used small RNA sequencing to characterize the miRNA cargo of EVs released from ALI-cultured bronchial epithelial cell line in response to infection with IAV. To our knowledge, this is the first study to complete small RNA sequencing of EVs released from IAV-infected bronchial epithelial cells cultured at ALI. RNA sequencing of EV miRNA released from IAV infected BCi compared to uninfected BCi revealed 5 miRNA to be upregulated with logFC > 0.6 (miR-122-5p, miR-155-5p, miR-146a-5p, miR-7-5p, miR-378a-3p) and 1 downregulated with logFC < -0.6 (miR-505-5p). A similar pattern of infection, EV miRNA release and gene expression was seen in both BCis and PBECs, suggesting the immortalized BCi cell line is a good model for studying influenza infection of the bronchial epithelium. Furthermore, our results suggest regulation of EV miRNA in response to IAV may be dysregulated in COPD, possibly contributing to the pathophysiology of the disease.

Analyses of the BCi cell sequencing data, mapped to miRBase, found that IAV infection significantly impacts on the miRNA EV profile. A logFC > 0.6 or < -0.6 cut-off was applied to identify miRNA altered above the minimal level of change of miRNA previously reported to have a significant impact on the biology of the cell (Kok et al., 2018). This revealed 5 miRNA to be upregulated for IAV infected BCi EVs with logFC > 0.6 (miR-122-5p, miR-155-5p, miR-146a-5p, miR-7-5p, miR-378a-3p) and 1 downregulated with logFC < -0.6 (miR-505-5p). A recent study reported EV microRNA expression profiles derived from A549 human lung cells in response to influenza A/H1N1pdm09 infection (Ge et al., 2022). There does not seem to be any overlap in the miRNAs detected with this study, most likely due to different cell type, infection conditions and EV isolation methods used. This highlights the sensitivity of the EV miRNA profile to different *in vitro* models and the importance of validating these findings *ex vivo*.

Sequencing identified miR-122 to be the miRNA with the biggest fold change in response to IAV. However, miR-122-5p was present at very low levels in BCi and PBEC EVs. This aligns with recent research that has reported miR-122-5p to be predominantly detected in the liver (Jopling, 2012). Interestingly other miRNA found to be altered in response to IAV, including miR-146a and miR-378a-3p, were also found to be enriched in EVs of HCV patients (Santangelo et al., 2018). This previous study suggested the shared common targets of these miRNA to have key roles in immune response and were markedly reduced following antiviral therapy (Santangelo et al., 2018).

The second most increased miRNA in BCi EVs in response to IAV was miR-155. An increase in cellular and EV miR-155 has been previously shown to be induced by IAV as well as in response to a wide range of other viruses such as rhinovirus and other non-viral stimuli including cigarette smoke and hypoxia (Gutierrez et al., 2015; De Smet et al., 2020; Woods et al., 2020; Meng et al., 2021). Furthermore miR-155-5p has been widely reported as a multifunctional miRNA enriched in cells of the immune system with a role in regulating a variety of biological processes including cell proliferation, apoptosis, inflammation and cell development (Hu et al., 2022). Given that miR-155 has been described as critical for immune regulation its presence may suggest that epithelial EVs may transfer regulatory signals beyond the epithelium to immune cells (Gutierrez et al., 2016). Furthermore, increased expression of microRNA-155-5p by alveolar type II cells has been shown to contribute to the development of lethal ARDS in H1N1 influenza A virus-infected mice (Woods et al., 2020). Therefore, miR-155 may also be responsible for viral-associated damage to the lung.

Our results demonstrated miR-146a to be the most abundant miRNA altered in response to IAV. However, despite miR-146a having a very small log_2_ fold change of 0.8, given the large abundance of this miRNA, even a small change may have a significant impact on target protein levels. Previous studies have shown miR-146a to be an abundantly expressed miRNA in various mammalian cell types and play a role in inflammation, differentiation, and function of adaptive and innate immune cells (Yuan et al., 2022). In addition, downregulation of miR-146a has been suggested to inhibit influenza A virus replication by enhancing the type I interferon response *in vitro* and *in vivo* (Zhang F. et al., 2019). Furthermore, CRISPR/Cas9-mediated deletion of miR-146a enhances antiviral responses in HIV-1 infected cells (Teng et al., 2019). In addition, macrophage-derived EVs enriched in miR-378a-3p have been shown to regulate cell death by blocking activation of the NLRP3/Caspase-1/GSDMD pathways in cardiomyocytes after myocardial infarction (Yuan et al., 2022).

Our data also add to the growing awareness of the co-operative functions of miRNA to fine-tune gene expression (Martinez-Nunez et al., 2014). Strongly validated target genes shared by multiple miRNA altered in response to IAV included *FADD*, *SOCS1* and *NFKB*. These genes have previously been shown to be involved in the viral immune response (Thompson et al., 2011; Oltean et al., 2021). IAV has been shown to activate FADD to drive apoptosis of infected cells and protects the host (Oltean et al., 2021). *FLNA* was also identified as a target of three of the EV miRNA identified to be upregulated in response to IAV (miR-378a, mR-7 and miR-155). FLNA is an actin-binding protein previously shown to be involved in regulating multiple signaling pathways and involved in the IAV replication cycle (Sharma et al., 2020). These results further suggest a function for these EV miRNA in modulating the response to IAV. However, a limitation of investigating the function of miRNA through *in silico* analyses is that validation of all miRNA targets still remains incomplete and in some cases biased towards the most prevalent areas of research, such as cancer.

However, the gene identified to be targeted by the most EV miRNA was *EGFR*. Network analyses revealed *EGFR* to be targeted by four of the EV miRNA upregulated in response to influenza, including miR-155, miR-122, miR-7 and miR-146a. EGFR activation has been described as a double-edged sword in influenza infection. EGFR signaling has been shown to support tissue regrowth during respiratory infection (Mitchell et al., 2019). However, it has also been suggested to promote viral replication through increased virion uptake or suppression of cytokine expression (Mitchell et al., 2019). Therefore, further work is required to understand the function of miRNA regulation of EGFR in response to IAV, particularly in the context of COPD, as EGFR signaling is known to be dysregulated in COPD (Strickson et al., 2023).

EVs have been identified as a novel mechanism of intercellular signaling involved in COPD pathogenesis (Lee et al., 2018). Burke et al. recently reported altered profile of miRNA from EVs isolated from BALF of 20 patients with COPD compared to 15 well-matched healthy ex-smokers (Burke et al., 2022). This previous work characterized the EV miRNA profile in stable COPD patients but our present study suggests that alterations in EV miRNA, and miR-155 particularly, may also play a role in acute exacerbations of COPD (AECOPD) caused by viral infection. COPD exacerbations are associated with increased airway inflammation usually triggered by bacterial and viral infections (Kwak et al., 2016). Exacerbations triggered by viral infections are usually associated with hypersusceptibility to greater airway inflammatory responses, more severe symptoms, and delayed recovery compared to those without viral infections (Tu et al., 2021).

Serum levels of miR-146a have been shown to be down-regulated in AECOPD patients compared with stable COPD patients and healthy controls (Chen et al., 2018). Furthermore, in AECOPD patients, levels of miR-146a in AECOPD patients were negatively associated with inflammatory cytokines including TNF-α, IL-6, IL-8, and LTE-4 expression. Mohamed et al. described the use of poly (glycerol adipate-co-ω-pentadeca-lactone) (PGA-co-PDL) nanoparticles to deliver miR-146a for COPD treatments due to its ability to downregulate signaling components of human body receptors (Mohamed et al., 2019). Transfection with miR-146a-NP reduced *IRAK1* gene expression. IRAK1 regulates multiple pathways in innate and adaptive immune responses by linking several immune-receptor complexes to TNF receptor-associated factor 6 (TRAF6), leading to activation of immune signaling pathways.

MicroRNA-378 inhibits the inflammatory response by targeting TNF-α, which may be a potential therapeutic target for COPD (Zhang JL. et al., 2019). Furthermore a study screening serum miRNAs for potential biomarkers for COPD reported upregulation of miR-7 (Akbas et al., 2012). Despite previous studies reporting increased miR-155 and miR-146a responses in COPD compared to healthy controls this was not observed in this study. In fact, healthy PBECS had higher EV miR-155 response to IAV than COPD PBEC EVs. Furthermore the variation in the EV miRNA vs cell miRNA ratio supports mechanisms involved in selective miRNA packaging into EVs (Lee et al., 2019). In addition, our results suggest that much of the extracellular miRNA may not be associated with EVs. Indeed previous studies have suggested extracellular miRNAs are in the most part by-products of dead cells that remain in the extracellular space (Turchinovich et al., 2011). However, alterations observed in EV miRNA in response to IAV were not always reflected in non-vesicular miRNA suggesting a potential function of EV miRNA over non-vesicular miRNA. The mechanisms behind EV miRNA packaging are complex and investigating the potential mechanisms for this were beyond the scope of this study.

One of the strengths of this study is the use of ALI-cultured differentiated BCi epithelial cells and primary epithelial cells from both healthy and COPD patients that more closely mimics *in vivo* infection conditions. However, we recognize that a much larger sample size of the primary PBECs would have been beneficial to increase the power of the results especially given the heterogeneity and complexity of COPD. Furthermore, future work to investigate if these changes in EV miRNA can be detected in COPD patients *in vivo* are also required. EVs should be isolated from healthy and IAV patient biofluids, particularly BAL fluid and serum, though it will be challenging to obtain BAL fluid from influenza-infected patients. These EVs may be separated based on parent cell type given EVs have been shown to contain proteins from parent cell type for example EPCAM and TSPAN8 have been described as epithelial cell EV markers (Sun et al., 2020).

Another strength is that this study used small RNA sequencing to determine the complete small RNA profile of EV. Whilst again recognizing the limitations of a small sample size, the results of this work provide a useful basis for more detailed functional studies using the targets of interest. The reliance on bioinformatics inference of miRNA function without further experimental validation is also a limitation. However, these miRNAs were investigated by qPCR in EVs as well as cellular and non-EV samples. This was important to determine if changes in miRNA in response to influenza were EV-specific. A further limitation of this study is that EVs overlap in size to IAV and therefore are likely to be co-isolated using the isolation methods in this study. This makes it particularly difficult to do functional characterization of the EV miRNA cargo. Future studies potentially investigating other isolation methods for the separation of EVs or the use of neuraminidase inhibitors that prevent the release of viral particles from host cells, thereby halting the spread of infection may overcome this.

EV miRNA identified in this study may be used to develop immune‐modulating therapies that strengthen the host defenses against influenza viruses and reduce tissue damage associated with viral infections especially in those with immune dysfunction such as COPD patients. However, this requires further research to determine the efficiency of EV miRNA transfer and uptake as well as determining the recipient cell type. In addition, further functional studies are required to validate the function of these miRNA in IAV infection to ensure no deleterious effects are observed.

## Data Availability

The original contributions presented in the study are publicly available in the Gene Expression Omnibus (GSE297071). This data can be found here: http://www.ncbi.nlm.nih.gov/geo/query/acc.cgi?acc=GSE297071.
